# Challenge of Brazilian health researchers in the COVID-19 pandemic scenario

**DOI:** 10.47626/1679-4435-2022-830

**Published:** 2023-02-13

**Authors:** Stephanie von Stein Cubas Warnavin, Rafael Zancan Mobile, Juliana Lucena Schussel

**Affiliations:** 1 Programa de Pós-graduação em Odontologia, Departamento de Estomatologia, Universidade Federal do Paraná, Curitiba, PR, Brazil

**Keywords:** COVID-19, researchers, health personnel, COVID-19, pesquisadores, trabalhador da saúde

## Abstract

Advances in Brazilian science made the country reach the 13th position in the world scientific production, and, in 2020, o Brazil was responsible for 2.39% of the world scientific production, reaching the 11th position among the countries that most published about COVID-19. The aim of this study was to contribute to and reflect on the issue of health researchers and graduate students in the scenario of COVID-19 pandemic. This pandemic highlighted the importance of science in the outcome of public policies and the fragility of the research system in Brazil, where the workforce is mainly composed of graduate students, who often do not have ideal working conditions and are not included in the response plans to global public health emergencies. This text brings a reflection and a questioning on the role of health researchers and graduate students and reinforces the importance of discussing the work of researchers/scientists in a period of great uncertainty in society.

## Abstract (pt)

Os avanços na ciência brasileira alcançaram a 13ª posição na produção científica mundial, e, em 2020, o Brasil foi responsável por 2,39% da produção científica mundial, alcançando a 11^a^ posição entre os países que mais publicaram sobre a COVID-19. O objetivo deste trabalho foi contribuir e refletir sobre o debate dos pesquisadores e pós-graduandos da área de saúde no cenário pandêmico da COVID-19. A pandemia da COVID-19 evidenciou a importância da ciência no desfecho de políticas públicas e a fragilidade do sistema de pesquisa no Brasil, em que a maior força de trabalho se concentra em pós-graduandos, que muitas vezes não possuem condições ideais de trabalho e não estão inseridos nos planos de resposta às emergências globais de saúde pública. Este texto traz uma reflexão e problematização sobre o papel dos pesquisadores e pós-graduandos em saúde e reforça a importância de discutir a atuação de pesquisadores/cientistas em um período de grandes incertezas na sociedade.

## INTRODUCTION

Over the last decades, Brazil has experienced a period of stimulated growth in the graduate system, which has been consolidated both nationally and internationally. Advances in Brazilian science made the country reach the 13th position in world scientific production,^1^ and, in 2020, Brazil published 4,029 studies on COVID-19, being responsible for 2.39% of the world scientific production and reaching the 11th position among the countries that most published about the theme.^2^

Since most of the national scientific production occurs in universities, especially public ones, the growth in national science is directly related to graduate activities and to the development of the National Graduate Program (Programa Nacional de Pós-Graduação, PNPG). Under the responsibility of the Coordenação de Aperfeiçoamento de Pessoal de Nível Superior (CAPES), the PNPG, which synthesizes public policies to qualify *stricto sensu* graduate programs, involves proposals of guidelines, strategies, and goals with the purpose of leverage Brazilian science and internationalize graduate programs.^3^

To become a researcher/scientist, it is necessary to take a long academic pathway during higher education. Starting from the Scientific Initiation Institutional Program (Programa Institucional de Bolsas de Iniciação Científica, PIBIC) up to master’s degree, doctoral degree, and post-doctoral degree, years of dedication are required to consolidate the training of university professors and researchers/scientists. In Brazil, it is estimated that 80% of the scientific production is conducted by master’s and doctoral students.^4^

Academic production is still one of the most important indicators and has an influence on the evaluation of graduate programs by CAPES, and is also an important criterion for students entering and remaining in undergraduate programs, as well as for receiving research funding from promotion agencies. Professors/advisors internalized the pressure for “productivist” demands and also foresee the consequences of not meeting these demands in their working conditions. The demand for high levels of productivity has implications on other agents of academic life, especially graduate students, requiring exclusive dedication for them to conduct high-quality research, with potential impact on their publications, in a scenario of reduced number of scholarships and outdated values.

Araújo^5^ describes the pressure advisors place on students to meet deadlines and publish articles as coauthors, which often results in evasion and approval in selective processes based on production rather than on skills. This vicious cycle results in deterioration of graduate students’ mental health, as widely reported in the international literature. Additionally, Kuenzer & Moraes^6^ analyzed that, in order to obtain the degree within the required timeframe - 24 months for master’s degree and 48 months for doctoral degree (based on the goals of CAPES assessment, limiting the maximum number of installments paid to scholarship holders) -, actual students’ working conditions are neglected. Curricular pathway assumes an idealized graduate student, with sufficient intellectual autonomy.

Despite the demand for increased dedication from graduate students, the last adjustment of graduate scholarships by CAPES occurred in 2013.^7^ For over 8 years, graduate students’ grants were not adjusted for the period’s inflation, but they are required to be fluent in a foreign language, publish more, participate in national and international congresses and to tackle the personnel shortage in the work field, laboratories, and class rooms, which is allied to the context of budget contingency, resulting in shortage of professors and technical-administrative employees.

The discussion about the regulation of the researcher/scientist professor advanced with the Senate Bill (Projeto de Lei do Senado (PLS) 212/2015. However, with the contrary position of scientists and directors of university institutions, who argue that being a scientist is an occupation, not a profession, the Bill did not advance in the Senate. Regardless of the debate on the regularization of scientists as a profession, it is a fact that the activity performed by graduate students is a work with low wage/allowance despite the high qualification required, takes a long period of time (6 years, on average, considering master and doctoral degrees) and do not entitle students to labor rights, such as social security contribution, vacations, Christmas bonus, transportation and food allowance, and health insurance.

## HEALTH GRADUATE STUDENTS AND THE COVID-19 PANDEMIC

On January 30th, 2020, the World Health Organization (WHO) declared a global public health emergency, and, in Brazil, the first case of COVID-19 was confirmed on February 26th, 2020. Since then, investigations, especially in the health field, have been reformulated and/or adapted to understand the current situation. Therefore, health graduate students started also to face several difficulties, with increasing risks for the conduction of their research.

In March 2020, public universities, followed by private ones, interrupted their in-person activities, which had an impact on the functioning of graduate programs. The focus on the necessary investigations about the SARS-CoV-2 influenced many ongoing studies and discontinued others, due to the urgency in obtaining new knowledge on this disease that affected the entire world. Many investigations conducted within universities or that involved field actions, either for data collection, prevention, and health promotion in several areas or non-COVID-19 outpatient clinical trials, had to be postponed and/or reconsidered for the new scenario of health restrictions. However, investigations in the hospital environment and in essential health services had autonomy to continue their activities, amidst all the insecurity of uncertain times and little knowledge, at that time, about the novel coronavirus. Furthermore, several studies were initiated, associated with prevention, diagnosis, and treatment of COVID-19, in the areas of biosafety, transmission, diagnostic methods, clinical trials, and search for a vaccine.

In this scenario, health graduate students all over Brazil committed themselves to do what they know best: science. Brazil reached the 11th position worldwide in investigations on COVID-19 due to the effort and dedication of these thousands of graduate students who did not feel discouraged and continued working to find answers and provide scientific-based information to the entire population. However, graduate students working in the fight against COVID-19 were not included as a priority group in the first phase of the Brazilian National Immunization Plan (Plano Nacional de Imunização, PNI).^8^

Similarly to other health care professionals, health graduate students who are in direct contact with infected individuals or biological material are exposed to an increased risk not only for SARS-CoV-2 infection but also for all the psychosocial impact that the COVID-19 pandemic has caused.^9-15^

Strategies targeted at health graduate students are necessary in order to provide them with increased safety in the conduction of their research in the current pandemic scenario. Their non-inclusion as a vaccination priority group, scarcity of personal protective equipment (PPE), and lack of adjustment in scholarship allowance (which does not include additional compensation for unhealthy work, in addition to the fact that students do not contribute to social security and, thus, are not covered against occupational accidents) synthesize the reality of abandonment that these professionals have currently faced.

## DECHARACTERIZATION OF SCIENTIFIC RESEARCH IN BRAZIL

The COVID-19 pandemic reached the country at a time when the technology, science, and innovation sector faced a scenario of great fragility, due to drastic financial and budgetary cutbacks.

It is worth noting that, over the last years, there was a devaluation of institutions responsible for research promotion in Brazil: the National Council for Scientific and Technological Development Conselho Nacional de Desenvolvimento Científico e Tecnológico, CNPq), linked to the Ministry of Science and Technology, and CAPES, linked to the Ministry of Education. Considering recent history, 2015 was the year when the greatest amount of federal funds were devoted to the scientific sector; however, after this period, investments have started to decrease.^16^

According to the budget proposal for 2021, developed by the Federal Government and under analysis in the National Congress, the Ministry of Science and Technology received an investment of approximately BRL 2.7 billion, which may be blocked throughout the year.^16^ In 2020, the amount allocated in the Federal Budget was BRL 3.7 billion; whereas in 2019, this value was BRL 5.7 billion. These data show a continuous devaluation of Brazilian research, with recurrent budgetary cutbacks.

According to CAPES Ordinance no. 20/2020, dated February 2020,^17^ the criteria for granting Social Demand scholarships of Graduate Support Programs (Programas de Apoio à Pós-Graduação, PROAP) from March 2020 to February 2021 underwent changes, thus establishing an initial amount for each institution, based on the qualification of each program. Therefore, according to this amount, there are two main factors for appraisal: the Municipal Human Development Index (MHDI) of the municipality where the graduate program is located and program’s average degree factor from 2015 to 2018, a fact that caused insecurity in programs with regard to the number of scholarships.

In addition to all these negative impacts for Brazilian research, the year 2020 was also marked by the “freeze” of research scholarships, which blocked approximately 11 thousand CAPES scholarships, through Ordinance no. 34/2020 of CAPES,^18^ published in March. Given this scenario, the president of the Brazilian Society for the Progress of Science (Sociedade Brasileira para o Progresso da Ciência, SBPC), Ildeu de Castro Moreira, stated that, with the appearance of the pandemic, the country needed funds to ensure well-equipped laboratories and qualified professionals; however, what happened was dismantlement of several institutions, due to a series of resource cutbacks.^16^ Graduate students who receive a scholarship are able to dedicate themselves exclusively to graduation activities and, therefore, are able to achieve a better performance. Reducing the number of scholarships means reducing the workforce and, consequently, reducing the number of scientific investigations.

The discrepancy and the cutbacks in scholarships and funds, allied to several other factors, such as physical and mental stress, made researchers’ work even more arduous and challenging. Furthermore, there is the decharacterization of the scientist profession in Brazil, taking into account the time required for researcher’s training, the scarce availability of scholarships, and the search for opportunities in the labor market. It is necessary to recognize the work of Brazilian researchers so as to value these professionals, who promote the development of investigations and the country’s scientific progress.

## MENTAL HEALTH AND WOMEN’S SCIENTIFIC PRODUCTION

There are several factors surrounding scientists’ lives and, with COVID-19, the lack of support to these professionals became even more evident. In addition to concerns with their own health and with that of their relatives, many researchers are worried about future perspectives of employment. The partial report of a research conducted with almost 5,900 early career researchers in the United Kingdom exemplifies the mental health status of the scientific community. The report showed that nearly three fourths of respondents had low levels of well-being. The same study indicated that two thirds of researchers interviewed worried about finances and future plans.^19^

In addition to the challenges faced, there is an additional factor to be highlighted: competitiveness in graduate programs and between researchers - either professors or students. This competition initiated in the 1990s, through a CAPES regulation targeted at researchers’ qualification, which made graduate programs to undergo significant changes in their form of funding, management, and assessment, having an impact on researchers and on the very quality of knowledge production.^20^

This system to assess graduate programs may collaborate to their better development, which may be considered positive, but, when observing the human factor of this assessment, the impacts are not so promising. Due to this system, the scientific and academic community is constantly challenged and pressed to achieve goals, collaborating to the onset of social, physical and, especially, mental problems.^21^

The concern with the impact of training on the mental health of students/researchers is not a new theme and has always been an object of discussion. Even before the beginning of the pandemic, there was also a mental crisis in the academic community. A study developed by Evans et al.,^22^ for example, demonstrated that depression and anxiety rates in graduate students were six-fold higher than those observed in the general population.

Allied to all difficulties resulting from the current health crisis, the pandemic intensified social inequalities, considering the differences in the observed impact according to race, social class, and gender.^23-25^ Several recent studies address the effects of social reclusion imposed by COVID-19 on women’s lives. These studies point to the occurrence of precarious working conditions and loss of job and income, along with work overload due to care provision to family members and, in more serious situations, increased rates of cases of domestic violence and femicide during this period.^24,26,27^ Within the scientific context - which is still predominantly male -, the reality experienced by female researchers follows the same pattern found in the studies.

With the closure of schools and the other social isolations measures, mothers and fathers became totally responsible for the care provision and the pedagogic process of their children, in addition to attempting to fill the absence of other social and affective relationships that were temporarily suspended. All this had an unequal impact on women’s lives and, for mothers of the scientific community, directly affects their academic performance, as well as on their physical and mental health. A decrease was observed in the number of articles published by women and, conversely, there was an increase in the number of publications authored by men,^28^ even in the pandemic period.^10^ This generates difficulties for women to obtain funding for their research and, therefore, for them to progress professionally. This process affects not only women’s academic lives, but the very diversity of knowledge production, which is already predominantly male, white, Western, and heterosexual.

## CONCLUSIONS

The COVID-19 pandemic highlighted the importance of science in the outcome of public policies and the fragility in the research system in Brazil, where the workforce is mainly composed of graduate students, who often do not have ideal working conditions and are included in the plans of response to global public health emergencies.

This text brings a reflection and a questioning on the role of health graduate students, and we know that there are no great expectations for this debate to reach a fair solution in time. However, we reinforce the importance of discussing the work of researchers/scientists in a period of great uncertainty in society.
